# Regulatory science of natural products

**DOI:** 10.1007/s11418-022-01639-w

**Published:** 2022-07-23

**Authors:** Yukihiro Goda

**Affiliations:** National Institute of Health Sciences, 25-26 Tonomachi 3-chome, Kawasaki-ku, Kawasaki, 210-9501 Japan

**Keywords:** Regulatory science, Quality assurance, Herbal medicines, Natural food additives, Food contaminants, Borderline products

## Abstract

Foods and pharmaceuticals play key roles in public health and welfare and ensuring that these products meet their quality assurance standards is a top priority in health and medical care. Quality assurance of natural products is essential in pharmaceutical sciences because the outset of a medicine is a natural, crude drug. Regulatory science underpins scientific regulations and is closely related to the quality assurance of foods and pharmaceuticals to ensure their safety and efficacy. During my time at the National Institute of Health Sciences, Japan, from 1986 to present, the regulatory science of natural products has been my main research focus. This review discusses 24 studies related to the regulatory science of natural food additives, 26 related to foods, 23 related to borderline products, 16 related to illicit psychotropic mushrooms, plants, and agents, and 57 related to herbal medicines. In later sections, the regulatory science for ethical Kampo products with new dosage forms and herbal medicines that use Kampo extracts as active pharmaceutical ingredients are discussed. My experience from the early twenty-first century in research projects on the bioequivalence of Kampo products and the development of ephedrine alkaloid-free Ephedra Herb extract demonstrate that regulatory science is crucial for developing new drugs.

## Introduction

Typical pharmaceutical studies evaluate the quality, safety, and efficacy of medicines, which contributes directly to public health and welfare; indeed, the quality assurance of products is a philosophy that forms the foundation of pharmaceutical sciences. At the outset of developing a medicine, we begin with the natural, crude drug. The elimination of substandard products and assuring the quality of crude drugs used in medical treatment is the basis of pharmacognosy and pharmaceutical sciences, which have developed and expanded alongside progress in medical science and medicines. For example, pharmaceutics and pharmaceutical analytical chemistry are the sciences directly responsible for the quality assurance of medicines, and hygienic chemistry is related to both the quality assurance of medicines and public health.

I belong to the National Institute of Health Sciences (NIHS), Japan. The institute was established in 1874 as the Tokyo Drug Control Laboratory and later renamed the Tokyo Institute of Hygienic Science in 1887. Nowadays, the NIHS regulates the products created by science and technology to ensure that they truly benefit the general public through scientific regulation. This ensures harmony between scientific technology and humanity, mostly in the field of pharmaceutics and in foods and chemicals present in our living environment. Therefore, the science implemented by the NIHS is called “regulatory science”, which is closely related to quality assurance of pharmaceuticals and foods to ensure their safety and efficacy. In this report, I will introduce the studies on the quality, safety, and efficacy of natural products that I have performed in the NIHS from 1986 onward.

## Regulatory science of natural food additives

My career began as a researcher in the Division of Food Additives in the NIHS. Initially, I focused on the coloring constituents of natural food colorants because they are used as commercial colorants without information on their main coloring constituents or their structures. This information is essential to define them in regulations. Structural identification of the coloring constituents was conducted for madder [1–3], safflower yellow [4, 5], hibiscus [6], *Monascus* yellow [7], purple sweet potato [8, 9], paprika [10], purple radish [11, 12], and annatto [13]. A new analytical method for bixin, the main coloring constituent of annatto in foods, was developed using supercritical fluid extraction and supercritical fluid chromatography [14].

The main pigment constituents of the color *Monascus* are the azaphilone compounds, monascorubrin and rubropunctatin, and their amino acid units in commercial *Monascus* pigments are the D- and L-forms [15]. For paprika, we developed an analytical method using HPLC [16] and investigated the photostability of the coloring constituents [17]. In addition, we studied the main coloring constituents of alkanet [18] and found that the shikonin isomer (*R*-configuration at the hydroxy group in the side chain) was more abundant than the alkannin isomer. This result suggested that the origin of the color was not *Anchusa officinalis*, as was shown in the official food additives list. Our chemical and genomic analyses confirmed this fact [19], although alkanet was deleted from the list in 2011.

Along with the identification of coloring constituents in natural food colorants, we performed the Ames test and found that madder derived from *Rubia tinctorum* showed mutagenicity. The mutagenic constituents isolated from the roots are anthraquinones and mollugins. Studies on the structure-mutagenicity relationships of anthraquinones revealed that the greatest activity was exhibited by 1,3-dihydroxyanthraquinones possessing methyl or hydroxylmethyl groups on carbon 2 (rubiadin and lucidin, respectively) [20]. The mechanism of mutagenicity of anthraquinones was elucidated by identifying adducts formed by the reaction of purine bases with lucidin [21]. Based on these data, animal studies [22–24] were performed and the use of madder as a colorant in foods was prohibited by the Ministry of Health Labor and Welfare (MHLW) in 2004.

## Regulatory science of foods

In 1988, cases of eosinophilia-myalgia syndrome (EMS) associated with the consumption of L-tryptophan products were observed in the USA, where most of the patients had consumed products derived from bulk L-tryptophan powder. Several groups were engaged in characterizing contaminants in tryptophan and the NIHS group found three HPLC peaks significantly associated with EMS that were designated as UV-5, UV-15 and UV-28 [25]. Smith et al. determined the structure of the compound that eluted in UV-15 (designated as peak E) as 1,1-ethylidenebis(l-tryptophan) [26]. The amount of compound from the UV-5 peak was small (approximately 0.01% by weight with respect to the total) and several laboratories competed to identify it. Finally, we identified the compound as 3-anilino-l-alanine [27]. Although successive studies have been performed to clarify the cause of EMS, an animal model has been difficult to establish and is not yet available.

Regulatory science for genetically modified (GM) foods is a main research subject, and I worked as a section chief of the food division in NIHS from 1996 to 2001. First, we compared the contents and composition of secondary metabolites of GM and non-GM soybeans and reported that they were substantially equivalent [28]. Subsequently, a joint research project with Dr. Hino of the National Food Research Institute in the Agriculture, Forestry and Fisheries Ministry for the development, detection, and determination of GM foods was started. We established a quantitative polymerase chain reaction (PCR) method for five lines of GM maize and GM soy by using constructed plasmids as reference molecules for GM maize and soy [29, 30]. Namely, the molecules contain the DNA sequences of a specific region found in each GM lines, such as cauliflower mosaic virus 35S promoter and nopaline synthase terminator, and the endogenous DNA sequences of maize (*zSSIIb*) or soy (*Le1*). To detect non-approved GM foods, we developed a method for detecting recombinant DNA (rDNA) from GM maize [31, 32], GM papaya [33], and GM potatoes [34, 35], and we also estimated the GM content in soybean material in tofu [36]. Furthermore, we first reported that unexpected rDNA was often detected in maize grains (not seeds) because of anemophily; therefore, quantitative detection of recombinant DNA in a certain number of fractured maize grains is needed to deduce the ratio of GM maize controlled by identity preserved handling, considering Mendelian characteristics and airborne pollution [37].

In 1996, a cow died when it ate a withered stem of a moroheiya plant (*Corchorus olitorius*) bearing ripened pods containing mature seeds, and strophanthidin (SP) was subsequently identified in the animal’s heart. To clarify the reason for the toxicity and allow the safe use of moroheiya as a vegetable, we analyzed samples and products containing moroheiya. We found that well-matured seeds contained large amounts of SP glycosides and pods also contained a small amount. However, no cardiac glycosides were detected in fresh green leaves, green stems, shoots, or pods, which are used as vegetables [38]. Furthermore, we did not detect cardiac glycosides in moroheiya health food products [39]. Along with the development of analytical methods, the cardiac glycosides in ripe seeds were isolated and the structure determined, and we isolated 11 compounds, including five SP glycosides, four digitoxigenin glycosides, a cannogenol glycoside, and a periplogenin glycoside, of which three were new compounds [40]. In our analysis, four SP glycosides (erysimoside, olitoriside, corchoroside A, and helveticoside) and two digitoxigenin glycosides (coroloside and glucoevatromonoside) were identified as the main cardiac glycosides. Gluco-(1 → 6)-olitoriside and olitoriusin, which were reported as the main cardiac glycosides by Mahato et al., [41] were not detected [40]. Finally, the acute oral toxicity of the isolated SP cardiac glycosides was tested in male ddY mice (6 weeks old). The LD_50_ values of mixtures of erysimoside and olitoriside (6:4) and coroloside and glucoevatromonoside (1:1) were > 500 mg/kg. Further toxicity testing could not be performed because the amount of isolated cardiac glycosides was insufficient [42].

Mycotoxins produced by fungi are a common hazard in food safety. We developed new analytical methods for aflatoxins in foods using multifunctional columns without harmful solvents [43, 44]. The detection limit for each aflatoxin was 0.1 ppb, and this method was adopted as the official method of the MHLW. Furthermore, because the targets of the method are easily expanded to herbal medicines, the method was also adopted in the World Health Organization guidelines for the analysis of aflatoxins in herbal medicines [45].

The bioactive constituents of foods are important for food safety and efficacy. During the analysis of the tertiary functions of vegetables and spices, flavonols that inhibited histamine release from RBL-2H3 cells induced by antigen stimulation were isolated from watercress (*Nasturtium officinale*) [46]. In collaboration with Prof. Ebizuka’s group at the University of Tokyo, we also investigated recombinant human lanosterol synthase inhibitors from taro (*Colocasia esculenta*) and found that digalactosyl diacylglycerols and monogalactocyl diacylglycerols are active constituents [47]. Therefore, we synthesized their derivatives and the structure-inhibitory activity studies revealed that digalactosyl diacylglycerols with two myristoyl groups at positions *sn*-1 and *sn*-2, with an oleoyl group at the *sn*-1 position, showed the most potent activity [48]. Laurel (*Laurus nobilis*) is one of the most widely used spices. We found its potent inhibitory activity against lanosterol synthase and isolated its constituents to obtain four flavonols and six sesquiterpenes. Of these, eight compounds had moderate inhibitory activity, and eremanthine, a sesquiterpene, was the most potent [49]. In addition, we screened Brazilian herbs for inhibitory activity against lanosterol synthase, and *Paffia paniculata*, *Trichilia catigua*, and *Maytenus ilicifolia* showed the most potent activity [50].

## Regulatory science of borderline products

The Division of Pharmacognosy, Phytochemistry, and Narcotics in the NIHS regularly handles borderline products between foods and drugs. Even after moving to this division in 2001, the quality of health food products, some of which are made from herbs, was still an object of research. A notification from the director of the Department of Food Safety of the MHLW stated that methods of guaranteeing the origin of products was the top priority to ensure the safety of health food products. During the course of our research on the origins of natural products using morphological, genomic, and chemical analyses, we found that the ingredients of some of the following health food products did not originate from the material listed on the label: chondroitin sulfate [51], white kwao keur (*Pueraria candollei* var. *mirifica*) [52], black cohosh [53, 54], haru-ukon (*Curcuma aromatica*) [55], bilberry [56], ginkgo leaf [57], chaste berry [58], and herbal materials for foodstuffs, such as Isodonis Herba and *Isodonis* extracts [59] and *Sida* products [60]. The average ratio of wrongly labeled products, including those with constituents from the stated origin but containing adulterants, to correctly labeled products was approximately one-third [61]. The results tested by the National Consumer Affairs Center of Japan for health food products of chondroitin sulfate and α-lipoic acid also showed a similar ratio in 2008. Furthermore, chromatographic analyses revealed that the content range of active ingredients in health foods, such as bilberry [56, 62], vine leaf [63], ginkgo leaf [57], and chaste berry [58], is wider than that of pharmaceutical products.

In Japan, many health food products are sold as tablets and capsules and their correct disintegration can be tested as an indication of quality. Our studies, in accordance with the Japanese Pharmacopoeia (JP) for ginkgo leaf and chasteberry products, showed that eight pharmaceutical products on the European market completely disintegrated within the defined test time, 20 min for capsules, 30 min for uncoated tablets, or 60 min for coated tablets; however, seven of the 18 tested herbal products distributed as health foods in Japan did not. Among the products that did not disintegrate properly, some remained intact after incubation in water for 60 min [64]. This result suggests that the quality of health food products is poor at a pharmaceutical level.

The borderline between foods and pharmaceuticals is judged by three main factors in Japan: the nature of the raw material (ingredient), claims of medicinal effects, and the directions for drug-like dosage and administration. If a health product intentionally contains an ingredient regarded as “raw materials exclusively used as pharmaceuticals” (RMEPs), it is controlled by the Pharmaceutical and Medical Device Law (formerly the Pharmaceutical Affairs Law). This means that health food products containing RMEPs are illegal in Japan and the seller is punished by the Pharmaceutical and Medical Device Law. The judgement standards for regarding ingredients as RMEPs based on their nature are publicly available as notification No. 243 in 2001 from the Director-General of the Pharmaceutical and Food Safety Bureau in the MHLW [65]. According to this notification, the content of toxic alkaloids is a judgement standard for RMEPs. Food companies can ask the MHLW whether a raw material is classified as a RMEP. However, companies sometimes sell a borderline product containing a raw material before or without the official judgement classifying the raw material as a non-RMEP.

It has been reported that Shatavari, a famous Ayurvedic materia medica originating from *Asparagus racemosus*, contains pyrrolo[1,2-a]azepine alkaloids, such as asparagamine A [66], which are also widely distributed in plants in the genus *Stemona*. Most members of the *Stemona* genus have tuberous roots, and the same vernacular names are sometimes used in local markets in Southeast Asia, even for representatives of other plant families because of their similar shapes. Our genomic and chemical studies of Shatavari plant materials and products [67, 68] revealed that *A. racemosus* materials and products did not contain such alkaloids and that the isolation of asparagamine A could have resulted from the misidentification of plants belonging to the *Stemona* genus as *A. racemonus*. Based on these results, Shatavari was reclassified from a RMEP to a non-RMEP in 2011.

Even though senna leaves are RMEPs, the stems are not, possibly because of the content of sennosides. Therefore, senna stems are used as ingredients in health food products. However, in some cases, health food products contain sennosides in medicinal quantities, which is thought to be due to the illegal use of the leaf rachises rather than the stems. Inspectors can find leaf rachises in other products consisting of non-powdered plant tissues such as tea bags. However, when the product is in powder form, it is extremely difficult to distinguish the different parts of plant tissues. To overcome this problem, we developed a morphological method for distinguishing powdered senna leaf rachises from powdered senna stems using microscopy [69].

β-Carboline alkaloids, such as harmine and harmaline, may have psychotropic activity due to monoamine oxidase inhibition. If raw materials contain these compounds, they could possibly be RMEPs. The origin of passionflowers is defined as *Passiflora incarnata* in the European Pharmacopoeia. However, in the Brazilian Pharmacopoeia, the origin is defined as *P. alata* and Pereira et al*.* suggest that *P. edulis* is sometimes used as a substitute [70]. In the list of non-RMEPs in Japan, the description is merely “passionflower”, which has not been corrected until now. Normally, we would regard “passionflower” as referring to the whole of the *Passiflora* genus. However, it has been reported that *P. incarnata* contains β-carboline alkaloids. Consequently, we investigated the source plants of 14 passionflower products available through the internet and their chemical compositions. The genetic and analytical results suggested that these samples were derived from *P. edulis* or *P. incarnata* and did not contain harmine or harmaline. In addition, one-third of the package indications did not show the true origin of the product. Indeed, the origin of some products that indicated their origin as “Passionflower (*Passiflora incarnata*)” were in fact derived from *P. edulis* [71].

During our studies on borderline plants, we investigated the constituents of dried rhizomes of *Neopicrorhiza scrophulariiflora* which is used as a traditional medicine in China, Tibet, Nepal, and India. We did not find any alkaloids or toxic compounds, although we did identify several new iridoid, cucurbitacin, and phenylpropanoid glycosides [72, 73]. Therefore, *N. scrophulariiflora* is thought to be a non-RMEP species.

## Regulatory science of illicit psychotropic mushrooms, plants, and agents

At the turn of the century, magic mushrooms (MM), which contain psychotropic compounds, such as psilocin and psilocybin, became popular in illicit drug markets. To clarify and define MM, we investigated the internal transcribed spacer region of the rRNA gene of *Psilocybe* and *Panaeolus* mushrooms and developed genetic discrimination methods for non-psilocybin mushrooms from MM [74]. We then developed a TaqMan PCR assay for the first screening identification of MM [75, 76]. Before the control of MM by the Narcotics and Psychotropics Control Law, we needed to supply psilocin and psilocybin as the determination standards for narcotics offices. Therefore, we developed a concise large-scale synthesis method [77].

After the regulation of MM, fly agaric-related products entered the Japanese illicit drug market. Genomic analyses showed that the origin was *Amanita muscaria* or *A. muscaria* var. *persicina*. Component analyses by LC–MS revealed that some of them contained harmine derivatives of which the deduced origin is an adulterated plant, *Peganum haramala*, and/or synthetic adulterants such as *N,N*-disopropyl-5-methoxytryptamine [78]. Thereafter, magic mint (*Salvia divinorum*), which contains the non-alkaloidal hallucinogenic compound, salvinorin A, appeared in the market. We conducted a survey to determine whether commercial *Salvia* cultivars available in Japanese horticultural markets contained salvinorin A prior to the regulation of *S. divinorum*. Ultra-performance liquid chromatography-MS analysis showed that none of the tested cultivars contained salvinorin A [79] and in 2007, the regulation of *S. divinorum* as a designated substance by the Pharmaceutical Affairs Law was implemented.

*Voacanga africana* is a small tropical African tree and its root bark and seeds contain several alkaloids, including hallucinogenic compounds, such as ibogaine. After the regulation of *S. divinorum*, some products, which appeared to contain *V. africana*, were distributed in the market. Because there were no analyses of the alkaloids in the products and their botanical origin, we investigated these products and found that they originated from *V. africana* or its closely related species. We classified the products into two chemical types: ibogaine-type and tabersonine-type. Based on the alkaloid distribution in *V. africana*, ibogaine-type products were derived from root bark and tabersonine-type products were derived from seeds [80].

Kratom is a leaf that has been traditionally used in Thailand and Malaysia for its opium-like effects and coca-like stimulant effects. To clarify the species of commercial kratom available in Japanese markets, we performed internal transcribed spacer sequence analysis of rDNA and found that these products were derived from *Mitragayna speciosa* or closely related plants [81]. In addition, the PCR-restriction fragment length polymorphism (RFLP) authentication method [82] that we used is highly practical owing to its wide range of applications, high accuracy, and simplicity [81]. Based on these results, as well as the development of a LC–MS analytical method for mitragynine alkaloids [83], which are active constituents, kratom has been regulated as a designated substance since 2016.

Hemp (*Cannabis sativa*) is a well-known psychoactive plant and its cultivation and possession are prohibited by the Cannabis Control Law in Japan. Hemp seeds have been used as a food, bird feed, and crude drug. To exclude the possibility of germination, it is officially mandated that the seeds must be killed before their legal distribution. To judge seed viability, a germination test was used. However, the test required several days and could not be used for on-site inspection. To solve this problem, we developed a method to detect hemp seed viability using 2,3,5-triphenyl-2*H*-tetazolium chloride as a coloring agent coupled with endogenous respiratory enzymes. The test is useful because the testing time is less than 20 min and the assay principle would prevent erroneous decisions [84].

From the end of the first decade in the twenty-first century, a variety of psychotropic products under names such as “spice” and “herbal blends” that look like plant powders or dried leaves have become widespread in the Japanese illicit drug market. At the end of 2008, we first identified a synthetic cannabinoid analog, cannabicyclohexanol ((1*RS*,3*SR*)-3-[4-(1,1-dimethyloctyl)-2-hydroxyphenyl]cyclohexan-1-ol), as a new type of designer drug in herbal products [85]. Around the same time, a German group also detected and identified a synthetic cannabimimetic indole, JWH-018, as an ingredient in herbal products [86]. Immediately after the first identification of JWH-018, we found JWH-018 and cannabicyclohexanol together in another product on the market [87].

In Japan, these herbal products are mostly sold as incense and advertised as not for human consumption, although their labels indicate that they contain a mixture of several potentially psychoactive plants. Interestingly, when we performed LC–MS analysis of an incense product, there were peaks corresponding to synthetic cannabinoids [88]. Therefore, we surveyed the chemical constituents and analyzed the DNA sequences to identify their plant sources. Chemical analysis revealed that the product contained tryptamine derivatives, such as mescaline, and carboline derivatives, such as harmaline. DNA analysis showed that it contained at least three kinds of cactus, namely, *Coryphantha macromeris*, *Lophophora williamsii*, and *Peganum harmala*. There were no contradictions between the constituents and plant species. However, except for this case, all spice-like herbal products sold as incense contained one or more synthetic cannabinoids. In 2013, we published the identification of plant species of botanical materials in spice-like herbal products by DNA barcoding methods [89]. The results showed that most of the detected plant species were not known to have psychoactive effects and that they did not appear on packaging labels. The plant materials were included mainly as diluents for synthetic cannabinoids, and the content and constitution of synthetic cannabinoids were not related to the detected plant species. However, in a few products, DNA fragments from some potent psychotropic plants, such as *Cannabis sativa* and *Mitragyna speciosa*, were found along with their active natural constituents.

## Regulatory science of crude drugs and herbal medicines

### Kampo extract and products

In Japan, the total production of herbal medicines by the pharmaceutical industry was approximately 1.8 billion USD, accounting for 2.9% of the total production value of pharmaceuticals in 2018. Kampo is a traditional Japanese medical system and most crude drugs are used in Kampo extract products. Therefore, the standardization of herbal medicines in Japan is generally concerned with the standardization of Kampo extract products. Because a typical Kampo formula consists of 5–20 different crude drugs, controlling the quality of these crude drugs is an essential step in standardization.

Standardization is the most important aspect of pharmaceuticals because it helps to ensure the reproducibility of medical treatments. However, standardization at the crude drug stage with several components is difficult because the diversity of plant secondary metabolites and their content are influenced by several factors, including genetic diversity among species, subspecies, hybrids, cultivars, climate, environment of the production area, collection/harvesting time (season), methods of harvesting, and processing conditions. Therefore, it is recommended that crude drugs should be produced using good agricultural practice. Specific good agricultural practices have been established for 68 crude drugs in Japan as of 2022. In the next stage, the quality of crude drugs is controlled according to the standards set out in the pharmacopoeias. The Japanese Pharmacopoeia, 18th edition (JP18), includes monographs for 237 crude drugs (56 powders), 37 Kampo extracts, 37 crude drug preparations other than Kampo extracts, nine plant oils, and five essential oils. Although the standardization of crude drugs is difficult, following large-scale extraction, quality control of the extract is standardized within itself, which is easier than standardization of the corresponding crude drug. Therefore, we have continuously examined Kampo extracts to be included in the JP.

Prior to the publication of the Japanese Pharmacopoeia, 15^th^ edition (JP15), Kampo extracts were not regulated by the JP. When I was assigned as the division head of the Division of Pharmacognosy, Phytochemistry, and Narcotics in the NIHS, I decided that the division had to deal with Kampo products because most herbal medicines in Japan are Kampo products. To ensure the quality of Kampo products, standardization is crucial. Therefore, after I became chair of the Expert Committee of Natural Medicines in the JP Committee in 2003, the committee started projects for adding Kampo extracts to JP monographs. After engaging in regulatory scientific discussions based on scientific data, 11 monographs for Kampo dry extracts were newly listed in the JP15 including supplements 1 and 2; 17 were listed in the Japanese Pharmacopoeia, 16^th^ edition (JP16) including supplements 1 and 2; seven were listed in the Japanese Pharmacopoeia, 17th edition (JP17) including supplements 1 and 2; and two were listed in JP18. In Japan, 148 Kampo extract formulations have been approved for ethical use. However, the market share of the 37 listed Kampo extracts accounts for approximately 75% of all Kampo products, suggesting that approximately 70% of the total output of herbal medicines in Japan is standardized by the JP.

The development of efficacy evaluation methods for Kampo medicines is also vital. Because Kampo products have characteristic tastes, creating placebo controls with similar tastes is difficult. Using over-the-counter (OTC) Kampo products, we developed a new pharmacist-centered research system to evaluate the usefulness of Kampo medicines based on the results of the actual use trial by Dr. Shimizu [90]. We named our system actual use research (AUR). First, an explanatory meeting on our system was held. Subsequently, a pharmacist (or pharmacy) was contracted by the AUR Implementation Committee. The pharmacist invited their customers to participate in the AUR. After obtaining consent from the pharmacist and answering several questions, the participant purchased the test OTC drug and began to keep a daily use record of dosage and time of intake, disease condition and use of other drugs. After a predetermined number of days, or when the symptoms of the disease disappeared, the participant returned to the pharmacy, submitted the daily record to the pharmacist and answered a questionnaire evaluating the usefulness of the drug, in addition to some questions from the pharmacist. The participant then received a gratuity. Independent of the participant evaluation, the pharmacist evaluated the usefulness of the drug based on the information obtained from the interview with the participant at the second meeting. Then, the pharmacist submitted the daily record, the questionnaire from the participant, and the pharmacist’s evaluation to the AUR Implementation Committee [91]. We implemented AUR three times for Kamishoyosan [92], Kakkonto, and Choreito [91] products and more than 85% of pharmacists evaluated their efficacy against some symptoms experienced by patients.

Before 2008, 210 Kampo formulae were approved to produce OTC Kampo products by the internal assignments in the review by the Pharmaceutical Affairs Bureau of the MHLW and the internal regulation was opened to the public in a book entitled “Guidance for OTC Kampo Formulae (Ippanyo Kampo Shoho No Tebiki)”. To expand the number of formulae and change their labeling efficacy in response to the changes in disease construction, in 2003, we established a project team, including medical doctors who were Kampo experts. Based on the discussions of the project team, the MHLW organized an expert panel to discuss the OTC Kampo formulae in 2008. The expert panel discussions and revision of the regulation were performed step-by-step. Finally, the new regulation of OTC Kampo products with revised efficacy was completed by the MHLW as notification No. 0830-1 on August 30th, 2012, from the Director of the Evaluation and Licensing Division, Pharmaceutical and Food Safety Bureau in the MHLW. The regulation approved 294 Kampo formulae, several of which contained processed aconite root (PAR) as a component [93].

The taste of crude drugs has been regulated as a criterion for judgement by an expert committee on natural medicines in the JP. However, taste is an organoleptic property that is difficult to express objectively. To clarify the taste of each Kampo formula objectively, we started using a taste-sensing system. First, we investigated the taste of five Kampo formulae (Kakkonto, Shosaikoto, Shoseiryuto, Rikkunshito, and Ryokeijutsukanto) [94]. The taste-sensing system evaluated five taste factors (sourness, umami, bitterness, astringency, and sweetness) using seven biomimetic sensor probes. Comparing the tastes measured by human gustatory tests with those measured by the system, the results for sweetness were consistent because humans are extremely sensitive to sweetness. In contrast, no one reported experiencing umami in the human gustatory tests, even though the system showed significant positive values. We proposed that this might be because the concept of umami is not generally extended to Kampo extracts. For some other taste factors, the results were sometimes different and we speculated that a strong taste factor could mask other taste factors in humans. In addition, the biomimetic system did not evaluate the taste of pungency, because pungency is not a taste perceived by taste cells. This may also explain the differences between the system and the human tests.

Despite these results, we found that the system was useful for the faithful standardization of the five taste factors. In particular, in human gustatory tests, even identical formulae vary in taste when produced by different pharmaceutical manufacturers and it can be difficult to express taste in a standard way. However, because taste measured with the system is expressed digitally, the system is superior in terms of the ease of standardization and expressing a uniform taste objectively for each formula [95].

We compared the tastes of several Kampo formulae and their component crude drugs. The results for Kakkonto suggested that its taste was similar to that of Ephedra Herb [96]. In contrast, no component crude drug solely expressed the taste of Ryokeijutsukanto and its taste was the combination of the tastes of the component drugs [97]. In response to the expansion of OTC Kampo formulae, it was necessary to standardize PAR. However, the crude drug is very toxic; therefore, it was difficult to describe its taste by human gustatory tests. PAR is classified into four types based on how it is processed. Using the taste-sensing system, we determined the objective taste of PAR and proposed a method for distinguishing between PAR types [98]. The taste sensing system can also be used to distinguish similar crude mineral drugs, namely Kasseki (aluminum silicate dihydrate with silicon dioxide in the JP) and Huashi (talc in the Chinese Pharmacopoeia), for which similar Chinese characters are used [99].

## Regulatory science of crude drugs

The use of authenticated crude drugs is one of the most important issues for ensuring the quality of Kampo and conventional crude drug products. Our group developed an authentication method for *Eleutherococcus senticosus* rhizome based on PCR–RFLP and estimated the detection limit for its application as a purity test. The results of the validation tests suggested that the method could detect contaminants at a level of 5% with respect to plant weight [100, 101]. PCR–RFLP was also used as a purity test for Atractylodes Rhizome for detecting *Atractylodes lancea*. Our validation study of the test was performed in seven laboratories, of which two participants were new to genetic methods. None of the laboratories provided an incorrect answer, proving that the PCR–RFLP method was robust [102]. Asini Corii Colla (ACC; donkey glue) is a crude drug originating from donkeys and is often adulterated with substances from other animals, such as horses, cattle, and pigs. Therefore, we developed a PCR method based on the sequence of the cytochrome *b* gene for source species identification. DNA extracted from ACC blended with 0.1% cattle glue was applied to cattle-specific PCR and the cattle-specific amplicon was detected. This result suggests that species-specific PCR methods are useful for the simple and easy detection of ACC adulteration [103].

Identification tests must be developed to regulate crude drugs according to the JP or non-JP crude drug standards in Japan. For regulation, a simple, rapid method is preferred; therefore, thin-layer chromatography is often used. For thin-layer chromatography identification of Hedysari Radix, we selected 9-*O*-methyl-coumestrol as the fluorescent marker compound because it distinguishes Hedysari Radix from Astragalus Root [104]. For Junci Herba, we selected luteolin 3′,5-dimethylether as the fluorescent marker because it distinguishes Junci Herba from horsetail (Sugina) and Ephedra Herb [105].

Metabolomic analysis is effective for grouping plant species in crude drugs. Cistanche Herba was added to the JP from JP16 supplement 2; before adoption, the range of plant species was discussed and four species, namely *Cistanche salsa*, *C. deserticola*, *C. tubulosa*, and *Bioschniakia rossica* were listed as Cistanche Herba in the self-imposed standards of the Japan Kampo Medicines Manufactures Association. Historically, wild *C. salsa* and *C. deserticola* have been used and in recent years, cultivated *C. tubulosa* has been used in China. In Japan, *B. rossica,* has been used as a substitute for *Cistanche*. The metabolomic analyses of crude drugs from these four plant species revealed that crude drugs originating from *Cistanche* had similar chromatographic patterns and that the variety of components from *C. salsa* and *C. deserticola* was wider than that of *C. tubulosa*. In addition, apparent differences in metabolomes between *Cistanche* and *B*. *rossica* were observed [106]. Based on these data, the plant origins of Cistanche Herba was determined to be *C. salsa*, *C. deserticola*, and *C. tubulosa* in JP16 supplement 2, and *B. rossica* was separately regulated as Boschniakia Herba (Wa-nikujuyo) in the non-JP crude drug standards.

## Regulatory science of reference standards

Flavanone glycosides, such as hesperidin, naringin, and neohesperidin, are often used as HPLC marker compounds to distinguish crude drugs from citrus species. However, their C-2 position is racemized easily by heat treatment and the *2S* and *2R* forms are diastereomers. We developed a separation method using HPLC and revealed that naturally occurring flavanone glycosides in citrus species would be the *2S* form, whereas the decoction process for preparing the Kampo extract converted them to diastereomeric mixtures. In addition, we found that all the commercial flavanone glycosides investigated were sold as their diastereomers [107, 108].

Glycyrrhizic acid was selected as a marker compound in HPLC assays of Glycyrrhiza, some Kampo extracts, and related products; the JP specifies a glycyrrhizic acid reference standard (GA-RS). In 1991, Kanaoka et al. suggested that GA-RS contained an impurity named peak X. However, until JP16, the HPLC conditions of their assays did not separate peak X from that of glycyrrhizic acid. Thus, the glycyrrhizic acid content determined by using GA-RS included peak X. However, owing to progress in chromatographic science and column technology, the Expert Committee of Natural Medicines in the JP Committee decided to include the HPLC conditions that separated the two compounds in the JP17. The structure of peak X was determined as 3-[β-D-galactropyranuronosyl-(1 → 2)-β-D-glucopyranuronosyloxy]glycyrrhetic acid mainly by nuclear magnetic resonance (NMR) analyses. The content of peak X in some lots of GA-RS and its effect on the quantification of glycyrrhizic acid itself were investigated. The results indicated that the GA-RS distributed after 2004 could be used in the JP17 assay [109].

In the JP, only 10 marker compounds, including glycyrrhizic acid, have been prepared as JP reference standards (RSs) for herbal medicines because of the difficulty in obtaining high-purity natural compounds. Unlike synthetic pharmaceutical substances, crude drugs are mixtures of several compounds. Although it is necessary to choose a substance contained in herbal medicines at levels of 0.1% to several percent as the marker compound for the quantitative assay, the synthesis of these compounds is often difficult. Therefore, the marker compound would need to be separated from the natural materials and isolated to achieve sufficient purity; however, this preparation method is costly and laborious.

Furthermore, in many cases, the greatest impurity in substances of natural origin is water. The Karl Fischer method can be used to determine the water content precisely; however, this would consume valuable RSs in addition to their primary purpose. Therefore, in many cases, commercial reagents are used as RSs for HPLC assays, despite their absolute purity not being determined. Over the past two decades, quantitative NMR (qNMR) has drawn attention across many fields because it provides accurate quantitative values without an additional RS; the RS is the same as the analyte. In addition, qNMR with SI (The International System of Units) traceability is possible using an appropriate protocol. Therefore, qNMR qualifies as a method for evaluating the RSs.

Since 2008, our group, consisting of NIHS researchers and members from an NMR company, a reagent company, and a pharmaceutical company, has conducted research on accurate qNMR with internal reference substance (AQARI) to determine the purity of reagents used as RSs for HPLC assays in crude drugs and Kampo formulations in the JP [110]. Validation studies showed that when the target reagents had a molecular weight of approximately 300, a mass of approximately 10 mg, and were dissolved in 1 mL deuterated solvent, AQARI could determine the purity with an accuracy of approximately two significant digits [111]. Furthermore, we found that the handling of impurity signals from reference substances and targeted marker compounds, chemical shifts of reference substances, and peak unity of signals of targeted marker compounds are important factors in conducting qNMR measurements with the intended accuracy [112]. Based on these studies, AQARI has been adopted as a method for purity determination of four reagents (geniposide, paeonol, magnolol, and magnoflorine) used for RSs in HPLC quantitative assays in JP16 supplement 2.

Humidity affects the purity of the reagents. Using thermogravimetric analysis, we confirmed that the hygroscopicity of natural marker compounds, such as ginsenosides and saikosaponins, could alter the purity of the compounds by increasing the water content [113, 114]. Therefore, we investigated controlling the purity and hygroscopicity of these commercially available reagents. We found that humidity control before and during weighing is important for reproducible preparation and that an indication of the absolute amount (not purity value), which is not affected by water content, is important for sale, because the humidity conditions in which the customer keeps the product cannot be controlled [114]. We then determined the typical and optimal conditions for determining the purity of several hygroscopic reagents [115]. Based on our continuing studies, until the JP18, 19 reagents evaluated by qNMR were listed as RSs for the HPLC assays in 37 crude drug and Kampo formula extract monographs, and these reagents with purity values determined by AQARI were commercially available.

## Regulatory science with reference to contaminants and residues

Agrochemical residues in herbal medicines have become a major safety concern for health authorities, the pharmaceutical industry, and the public. Our group, together with the Hokkaido Institute of Public Health, developed analytical methods for organophosphorus pesticide residues and surveyed their content in 37 types of crude drugs [116–118]. Furthermore, we investigated the migration ratio of organophosphorus pesticides to Kampo products [119] and crude drug decoctions [120] because these pesticides are hydrophobic compounds. The results suggested that considerable amounts of pesticides were not extracted from the crude drugs and that some of the extracted pesticides disappeared during the decoction and drying processes. The maximum migration ratio in Kampo decoctions was 31% [119] and the final pesticide residue levels in the freeze-dried and spray-dried extracts were 10–14% and 2–3% of those observed in crude drugs, respectively [121]. These data suggest that the discussion and regulation of pesticide levels in final products are important for the safety of crude drug products.

The fumigant content is also a major concern for crude drug safety. In China, sulfur fumigation is performed on some crude drugs for bleaching, drying, and killing insects and bacteria. Therefore, we analyzed the content of sulfur dioxides for 31 types of crude drugs purchased from the Japanese market and sulfur dioxide levels of more than 1000 ppm were detected in three (Dioscorea Rhizome, Gastrodia Tuber, and Fritillaria Bulb) and more than 500 ppm were detected in seven (Pueraia Root, Lilium Bulb, Ginger, Asparagus Tuber, Platycodon Root, Mulberry Bark, and Forsythia Fruit) using the modified Rankine method [122]. Considering regulations for foods (e.g., a limit of 5000 ppm for dried gourd shavings), the contents were not toxic, but the taste of the decoctions changed. Decoctions from crude drugs with different sulfur dioxide contents were analyzed by our taste-sensing system and high sulfur dioxide contents reduced the intensity of umami and increased the intensity of astringency and bitterness, producing worse tastes [123].

## Regulatory science by microscopic examination

*Nicotiana tabacum* (Solanaceae) is the only species whose leaves can be marketed legally as tobacco according to the Japanese Tobacco Business Act. Nicotine, a major alkaloid produced by *N*. *tabacum* leaves, is regulated as a pharmaceutical by the Pharmaceutical and Medical Device Law. However, the use of *N*. *tabacum* stems as an excipient in pharmaceuticals is permitted because they contain only a small amount of nicotine. However, several reports have shown that a substantial amount of nicotine was detected in an OTC pharmaceutical product in which *N. tabacum* stems were used as excipients. Therefore, products containing *N. tabacum* stems could be contaminated with leaf material. We established a microscopy method to detect contamination of *N. tabacum* stem materials with leaves to obtain standard reference microphotographs for identification [124].

Microscopic examination of crude drug components is the traditional method for identifying the origin of biological materials. To identify components in a given mixture via microscopy, standard reference photographs of fragments derived from different organs and tissues of individual species are required. Even if these references are available, a highly observant eye is needed to compare the morphological characteristics found under the microscope with the available references and to identify the origins of the materials. Therefore, using other indices to support microscopic examination would greatly improve the accuracy of identification. To identify the crude drug Quanxie (dried scorpion), standard reference photographs were prepared for microscopic examination. In addition, because scorpions possess the remarkable property of fluorescing under ultraviolet light, two methods to identify the crude drug were established: fluorescence fingerprint analysis and microscopic fluorescent luminance imaging analysis [125].

## Regulatory science for new dosage forms of herbal medicines

Since the 1980s, no new ethical drugs consisting of herbal extracts have been approved in Japan. Moreover, even new dosage forms of ethical Kampo products have not yet been approved. This is because there is no official guidance for marketing authorization of an ethical natural medicine consisting of herbal extracts in Japan. Manufacturers do not challenge this without evaluating cost performance by using official guidance, even if they are seeds of a new drug. Because Kampo products are often used for elderly patients, new dosage forms are required in Japan’s aging society. Bioequivalence is the concept of checking the equivalence of the pharmacokinetics of a previously approved drug with those of a new drug. Therefore, in 2009, we started a research project to consider the bioequivalence of Kampo products.

The clinical effects of Kampo extract products are expected to be similar to those of the corresponding Kampo decoction, which is why Kampo extract products are approved as ethical drugs. We thought that a marker compound showing bioequivalence between a Kampo decoction and its corresponding product would be useful for evaluating the bioequivalence between new dosage forms and products that are already approved. First, we compared the bioequivalence of Kakkonto decoctions and the extract product using ephedrine, pseudoephedrine, puerarin, daidzein, liquiritin, paeoniflorin, and glycyrrhizic acid as marker compounds in human blood plasma after oral administration. Ephedrines showed sufficient statistical power for both the maximum plasma concentration and area under the plasma concentration–time curve to evaluate bioequivalence for more than 14 study participants [126]. In contrast, the other compounds did not show bioequivalence, possibly because of metabolic differences in individual intestinal bacteria, content differences as dietary constituents. or enterohepatic circulation [127].

We investigated Shoseiryuto decoction and its extract product and found that ephedrine, pseudoephedrine, asarinin, [6]-shogaol, gomisin A, and schisandrin could be used as markers for evaluating bioequivalence between new dosage forms and those that are already approved [128, 129]. Our evaluation of Hachimijiogan showed that benzoylmesaconine and 14-anisoylaconine may be appropriate marker compounds [130]. Next, we performed a bioequivalence test between Kakkonto extract granules, which were already approved, and Kakkonto extract tablets, which were a new dosage form, by using ephedrine and pseudoephedrine as markers. The pharmacokinetic parameters of the markers were similar following the administration of the Kampo extract products and the corresponding standard decoction, and thus these compounds were suitable as markers for evaluating bioequivalence between an already approved Kampo product and a new product [131]. Based on this data, we discussed the guidance for the bioequivalence of Kampo products with new dosage forms with MHLW and Pharmaceuticals and Medical Devices Agency (PMDA) members. The guidance was published by the Evaluation and Licensing Division in the MHLW on September 19th, 2021.

## Regulatory science for new ethical drugs derived from herbal medicines

I discovered that there was no guidance for the approval process of ethical drugs derived from herbal medicines in the early twenty-first century and I felt that this was detrimental to drug development. Therefore, I asked PMDA to assist us, but the proposal was rejected by the PMDA.

Ephedra Herb is defined as a dried stem of *Ephedra sinica* Stapf, *Ephedra intermedia* Schrenk et C. A. Meyer, or *Ephedra equisetina* Bunge (Ephedraceae) in the JP18. The material contains more than 0.7% total alkaloids (ephedrine and pseudoephedrine) and is widely known for its diaphoretic, antipyretic, antitussive, and anti-inflammatory properties [132]. Since ephedrine was determined as the principal component of the Ephedra Herb by Prof. Nagayoshi Nagai in 1885 [133], it has been believed that ephedrine alkaloids, including pseudoephedrine, methylephedrine, methylpseudoephedrine, norephedrine, and norpseudoephedrine, play a major role in its pharmaceutical actions [132]. However, ephedrine alkaloids present in Ephedra Herb induce side effects including hypertension, palpitations, insomnia, and dysuria. Consequently, Kampo medicines containing Ephedra Herb should be administered cautiously to patients with circulatory impairment, hypertension, or renal impairment, or who are physically fragile or elderly [134].

In 2011, the collaboration of Dr. Hyuga and Dr. Hanawa from the Oriental Medicine Research Center of Kitasato University (OMRC-Kitasato) with our group found a previously unknown pharmacological action of Ephedra Herb, which impaired hepatocyte growth factor (HGF)-induced cancer cell motility and growth by suppressing the phosphorylation of the HGF receptor (c-Met) [135]. The c-Met inhibitory activity of Ephedra Herb was independent of ephedrine alkaloids.

Furthermore, a collaboration between OMRC-Kitasao, Matsuyama University, and our group found that Ephedra Herb contains new flavonoid glycosides, herbacetin 7-*O*-neohesperidoside, and herbacetin 7-*O*-glucoside [136], and their aglycones inhibited HGF-induced cell migration and phosphorylation of c-Met [137]. Because these glycosides are hydrolyzed to herbacetin by the intestinal flora after oral administration, herbacetin is thought to be an active metabolite of herbacetin glycosides in Ephedra Herb. In addition, our collaborative studies revealed that herbacetin inhibited c-Met tyrosine kinase, along with tropomyosin receptor kinase A (Trk A), Aurora kinase, and Fms-like tyrosine kinase 3, and thus is a multikinase inhibitor [138]. Trk A is a nerve growth factor (NGF) receptor. NGF is an inflammatory mediator and NGF-Trk A signalling is involved in inducing pain and itching [134]. Therefore, we investigated the analgesic effect of herbacetin using the formalin test [139]. The results suggested herbacetin suppressed the second phase of formalin-induced pain in a dose-dependent manner. Namely, it functions as a nonsteroidal anti-inflammatory agent, indicating that herbacetin glycosides in Ephedra Herb may also contribute to the analgesic action.

Herbacetin has c-Met inhibitory activity and analgesic effects and Ephedra Herb has ephedrine alkaloid-independent pharmacological actions. However, the c-Met inhibitory activity of Ephedra Herb could not be explained by herbacetin glycosides because their content was not as high (approximately 0.1%) and they had no direct effect on c-Met. Therefore, the non-alkaloid fraction of the Ephedra herb was presumed to contain other ingredients with c-Met inhibitory activity. It was previously thought that removing ephedrine alkaloids from Ephedra Herb would abrogate its pharmacological actions [132]. However, our findings contradicted this and led to the idea that ephedrine alkaloid-free Ephedra Herb extract (EFE) could be used as a new natural medicine with fewer adverse effects than Ephedra Herb extract (EHE) itself. Furthermore, from a practical point of view, I thought that if clinical research suggested that EFE was a potential new natural medicine, the PMDA should discuss a method of assuring its quality with us. Therefore, with help from Tokiwa Pharmaceutical Co., we prepared EFE using cation-exchange column chromatography to eliminate the alkaloids. Chromatographic analyses revealed that the ephedrine alkaloid content in EFE was less than 10–0.05 ppm [140]. EFE exerts analgesic, anti-influenza, and anticancer effects in the same manner as EHE [141], and the pharmacological activity of EFE has been summarized in a book chapter [134].

In 2016, Dr. Odaguchi (OMRC-Kitasato) and our cooperative team performed a double-blind, randomized, crossover comparative study to evaluate the clinical safety of EFE (UMIN000022061) [142]. The results suggested that EFE was not inferior to EHE in terms of safety. Because EFE showed no toxicity in pharmacological studies [143] and the number of adverse events in the clinical study was marginally higher in the EHE-treated group than in the EFE-treated group, we concluded that EFE was safer than EHE [144].

We then started the quality evaluation and characterization of the fractions of EFE with biological activity [145]. The results suggested that the chemical properties of the active fractions indicated high-molecular mass condensed tannins and that they were mainly B-type procyanidins; however, a portion contained A-type procyanidins, including pyrogallol- and catechol-type flavan-3-ols as extension and terminal units. The ratio of pyrogallol- to catechol-type flavan-3-ols was approximately 9:2 and the average molecular weight based on the polystyrene standard was > 45,000. We named the active fractions Ephedra Herb macromolecule condensed-tannin (EMCT) and developed a gel permeation chromatography quality evaluation method for EMCT in EFE. In addition, we found that flavone C-glycoside can be used as a quality control marker for the manufacturing process of EFE as a crude drug preparation [146].

In 2020, the coronavirus disease 2019 (COVID-19) pandemic began. Because we found that EFE was a safe material and had antiviral activity, our group, represented by Dr. Odaguchi, applied for research expenditure for clinical studies (including phase 2) on COVID-19 patients. Expenditure was allocated, and before the clinical study, we started to discuss how to ensure the quality of EFE with the PMDA. The discussion is the first trial of natural medicine for new ethical drugs since the 1990s in Japan and the results provided much information to guide the approval process for ethical drugs derived directly from herbal medicines, although we cannot disclose the details publicly.

## Conclusion

Regulatory science is the foundation of scientific regulation. During my research period in the NIHS, studies of the scientific regulation of synthetic substances, including dyes, illegal drugs, pharmaceutical excipients, medicinal products, and pure natural product chemistry, were carried out in addition to those on the regulatory science of natural products. However, these studies are not addressed in this review.

In my opinion, the most important aspect of regulatory science is its practical purpose. First, we consider the safety and benefits for the general public. The government make regulations for public health and welfare, even if there is insufficient scientific data, in which case, regulatory scientists must draw deductions from existing data. Should regulatory scientists obtain new data that invalidates an earlier decision, we should share this with the public in an effort to propose a new regulation. The cases of the madder food color and Shatavari Ayurvedic medicine are typical examples. In later sections, I discuss the regulatory science for ethical Kampo products with new dosage forms and new herbal medicines based on Kampo extracts as the active pharmaceutical ingredient. My experience with these research projects on the bioequivalence of Kampo products and development of EFE demonstrates that regulatory science is necessary even for the development of a new drug. As regulatory scientists, we must be aware of the balance between safety and benefits. Viable regulation is needed for harmony between scientific technology and humanity based on regulatory science.
